# Current Applications of Chatbots Powered by Large Language Models in Oral and Maxillofacial Surgery: A Systematic Review

**DOI:** 10.3390/dj13060261

**Published:** 2025-06-11

**Authors:** Vincenzo Ronsivalle, Simona Santonocito, Umberto Cammarata, Eleonora Lo Muzio, Marco Cicciù

**Affiliations:** 1 Department of Biomedical and Dental Sciences and Morphological and Functional Imaging, University of Messina, 98122 Messina, Italy; vincenzo.ronsivalle@hotmail.it; 2 Department of General Surgery and Surgical-Medical Specialties, School of Dentistry, University of Catania, 95124 Catania, Italy; umberto.cammarata0905@gmail.com (U.C.); marco.cicciu@unict.it (M.C.); 3 Department of Clinical and Experimental Medicine, University of Foggia, 71122 Foggia, Italy; eleonoralomuzio@gmail.com

**Keywords:** artificial intelligence, chatbots, conversational agents, large language models, natural language processing, oral and maxillofacial surgery, dental surgery, ChatGPT, Microsoft Copilot, Google Gemini

## Abstract

**Background/Objectives:** In recent years, interest has grown in the clinical applications of artificial intelligence (AI)-based chatbots powered by large language models (LLMs) in oral and maxillofacial surgery (OMFS). However, there are conflicting opinions regarding the accuracy and reliability of the information they provide, raising questions about their potential role as support tools for both clinicians and patients. This systematic review aims to analyze the current literature on the use of conversational agents powered by LLMs in the field of OMFS. **Methods**: The review was conducted following PRISMA guidelines and the Cochrane Handbook for Systematic Reviews of Interventions. Original studies published between 2023 and 2024 in peer-reviewed English-language journals were included. Sources were identified through major electronic databases, including PubMed, Scopus, Google Scholar, and Web of Science. The risk of bias in the included studies was assessed using the ROBINS-I tool, which evaluates potential bias in study design and conduct. **Results**: A total of 49 articles were identified, of which 4 met the inclusion criteria. One study showed that ChatGPT provided the most accurate responses compared to Microsoft Copilot (ex-Bing) and Google Gemini (ex-Bard) for questions related to OMFS. Other studies highlighted that ChatGPT-4 can assist surgeons with quick and relevant information, though responses may vary depending on the quality of the questions. **Conclusions**: Chatbots powered by LLMs can enhance efficiency and decision-making in OMFS routine clinical cases. However, based on the limited number of studies included in this review (four), their performance remains constrained in complex clinical scenarios and in managing emotionally sensitive patient interactions. Further research on clinical validation, prompt formulation, and ethical oversight is essential to safely integrating LLM technologies into OMFS practices.

## 1. Introduction

Recently, technological advancements in artificial intelligence (AI) have contributed to the growing integration of AI-based chatbots, including conversational agents powered by large language models (LLMs), within the medical and dental fields [1,2,3]. The concept of chatbots originated in the 1960s with ELIZA, developed in 1966 by Joseph Weizenbaum, one of the first natural language processing (NLP) programs designed to simulate psychotherapeutic dialog [4]. Subsequently, in 1972, Kenneth Colby developed PARRY, a chatbot simulating a paranoid schizophrenic patient, representing a significant advancement over ELIZA. In the following years, other chatbots emerged, such as ALICE (Artificial Linguistic Internet Computer Entity) in the 1990s and Mitsuku (2005), which employed rule-based algorithms for more sophisticated interactions [5,6]. The 2010s saw the introduction of voice-activated assistants like Apple’s Siri (2011), Amazon’s Alexa (2014), and Microsoft’s Cortana (2014), integrating natural language understanding (NLU) and speech recognition, paving the way for current conversational agents based on LLMs [7]. A breakthrough occurred in November 2022, with the release of ChatGPT by OpenAI, an LLM-based chatbot capable of generating coherent, context-aware natural language responses across a wide range of topics. Since then, other advanced platforms have emerged, including Google Bard (now Google Gemini), Microsoft Bing Chat (now Microsoft Copilot), and Claude by Anthropic. These systems are known for their advanced conversational abilities and integration with search engines and professional environments [8]. While these systems are implemented as chatbot interfaces for ease of use, they are fundamentally based on powerful LLMs—deep learning models trained on vast corpora of unstructured text. Although chatbots primarily serve interactive dialog purposes, LLMs can also perform a wide range of tasks beyond conversation, such as text summarization, clinical documentation, and data extraction [9,10,11].

Within healthcare and dentistry, chatbots powered by LLMs are increasingly used to provide accessible information on topics including oral and maxillofacial surgery (OMFS) [12,13]. They offer both patients and professionals rapid access to seemingly accurate answers for various clinical and surgical inquiries [2,14,15]. For instance, patients may use chatbots to learn about treatment expectations, pre- and post-operative care, and surgical procedures [8,16]. However, none of the existing chatbots—such as ChatGPT (Open-AI), Google Gemini (ex-Google Bard), and Microsoft Copilot (ex- Microsoft Bing)—were created to offer medical services [8,16]. The use of chatbots in healthcare is still debatable, despite earlier research showing that ChatGPT, despite being trained solely on publicly available online data, correctly answers test questions from the United States Medical Licensing Examination (USMLE) more than 90% of the time [17,18].

Chatbots powered by LLM could assist physicians in analyzing large datasets and provide decision support mechanisms to improve the quality of healthcare services [18,19]. However, their use in the medical sector remains a subject of ongoing debate because their reliability and accuracy in addressing highly specialized topics, such as OMFS, are not consistently ensured [11,20]. Despite their growing use, the current literature lacks a comprehensive synthesis of how these technologies perform specifically in the domain of oral and maxillofacial surgery. Most existing studies present isolated evaluations without critical integration or comparison, and there is a need to better understand both their potential utility and inherent limitations in specialized clinical contexts. Moreover, there is limited consensus on their reliability and safety in complex medical scenarios, where human expertise remains crucial.

In a previous study, ten experienced surgeons evaluated how well three popular chatbots—ChatGPT, Google Gemini, and Microsoft Bing (now Microsoft Copilot)—performed on oral surgery-related queries, noting that ChatGPT gave more thorough and accurate responses than the other chatbots [21]. In a different study, the authors evaluated the quality of dental information produced by ChatGPT in the context of oral surgery, preventive dentistry, and oral cancer, concluding that professional supervision is necessary during the use of information generated by chatbots using LLMs, especially when consulted by patients, as there are still many fields of dentistry in which they present limited completeness and accuracy, such as oral surgery, post-therapy indications, and oral cancer [11]. The accuracy level and potential applications of these technologies demonstrated how the findings are frequently unclear and inconsistent in certain aspects [22,23].

Therefore, the aim of this review is to assess the current literature concerning the application of AI-based chatbots powered by LLMs in the field of OMFS, with a specific emphasis on evaluating their accuracy, clinical utility, and reliability in supporting surgical decision-making, highlighting the possible strengths and limitations of the available LLM technologies, and to provide possible future research perspectives that will facilitate the safe and accurate entry of these new technologies into daily clinical practice.

This review should be considered an initial synthesis of the evidence, reflecting the nascent state of research on LLM-based chatbots in oral and maxillofacial surgery. The limited number of available studies and their heterogeneity restrict the generalizability of conclusions. Nonetheless, this exploratory analysis offers valuable insights and underscores the urgent need for larger, methodologically rigorous studies to better understand the potential and limitations of this technology.

## 2. Materials and Methods

This systematic review was conducted according to PRISMA (Preferred Reporting Items for Systematic Reviews and Meta-Analysis) guidelines and the Cochrane Handbook for Systematic Reviews of Interventions to ensure transparency and rationality in the entire process. The systematic review protocol was registered in PROSPERO (International Prospective Register of Systematic Reviews) under the protocol number CRD420251016515. Although the protocol was formally submitted to PROSPERO on 21 March 2025, the registration was finalized later, on 21 May 2025. The literature search was conducted between 28 March and 8 April 2025.

The research question guiding this review was “In human subjects undergoing OMFS, what are the applications and limitations of conversational agents powered by LLMs, such as ChatGPT, Gemini, and Copilot, compared to conventional information sources or other LLM-based chatbot platforms, according to evidence from primary research studies published between 2023 and 2025?”.

### 2.1. Eligibility Criteria

Studies were selected based on the following Population, Intervention, Comparison, Outcome and Study type (PICOS) model:

- (P) Human subjects including patients, dental professionals, or laypeople;
- (I) LLM-based chatbots, such as ChatGPT (Open AI), Gemini (Google), and Copilot (Microsoft), that were implemented in OMFS domains, where they included both traditional oral surgery procedures and more extensive surgical interventions in the maxillofacial region;
- (C) Conventional approach for finding medical information (brochures or human experts) or LLM-based chatbots comparison;
- (O) Evaluation of the answers provided by AI-based Chatbots, in terms of accuracy, precision, readability, and response processing speed, in the field of OMFS;
- (S) Primary research articles published in English in peer-reviewed academic journals between 2023 and 8 April 2025 were included.

Primary research was defined as studies presenting original data, including clinical trials, observational studies, and experimental research. The narrowed publication window reflects the rapid emergence of LLM-based chatbots, beginning with the release of ChatGPT in late 2022.

The exclusion criteria were as follows: (1) articles that did not comply with PICOS; (2) review articles, editorials, and commentaries; (3) studies written in a language different from English; and (4) full text unavailability.

### 2.2. Search Strategy

A comprehensive literature search was conducted across PubMed, Scopus, and Web of Science. Google Scholar was used only as a supplementary source to identify potentially relevant studies not indexed in the primary databases. Reference list screening and gray literature were excluded to maintain a focused and reproducible search strategy, given the emerging and still limited body of indexed literature on chatbots in OMFS.

The search strategy was structured using a combination of controlled vocabulary and free-text terms. The following keywords and their synonyms were used:

(“oral surgery” OR “dental surgery” OR “maxillofacial surgery”) AND (“large language models” OR LLM OR ChatGPT OR GPT OR “generative pre-trained transformer” OR “pretraining language model” OR “AI language model” OR chatbot OR “natural language processing” OR NLP).

A detailed overview of the search terms and Boolean operators used is provided in Table 1.

### 2.3. Study Selection and Data Extraction

All records were imported into EndNote version 20 reference management software (Clarivate, Philadelphia, PA, USA) for systematic organization and removal of duplicates. The screening and selection process was conducted in two phases:

(1) Title and abstract screening: Two independent reviewers (U.C. and S.S.) evaluated the titles and abstracts of all records against the predefined eligibility criteria based on the PICOS framework. Records clearly not meeting inclusion criteria were excluded at this stage.
(2) Full-text assessment: For all studies that met the inclusion criteria or where relevance remained uncertain, the full texts were retrieved and independently reviewed in detail by the same two reviewers. Studies not meeting eligibility criteria upon full-text review were excluded, with reasons documented.

Any discrepancies that occurred during the screening process were resolved through evaluation by an independent third-party reviewer (M.C.).

To assess the inter-rater reliability between the two reviewers during the screening process, Cohen’s kappa coefficient (κ) was calculated. This statistic measures the level of agreement beyond chance, with values interpreted according to established benchmarks. The analysis was performed using common Statistical Package for Social Sciences (IBM SPSS v.29).

The data extracted included the characteristics of the articles (authors, country and year of publication), the conversational agents powered by LLMs (name and company), and the application (performance/efficacy/readability of the studies).

**Figure 1:**
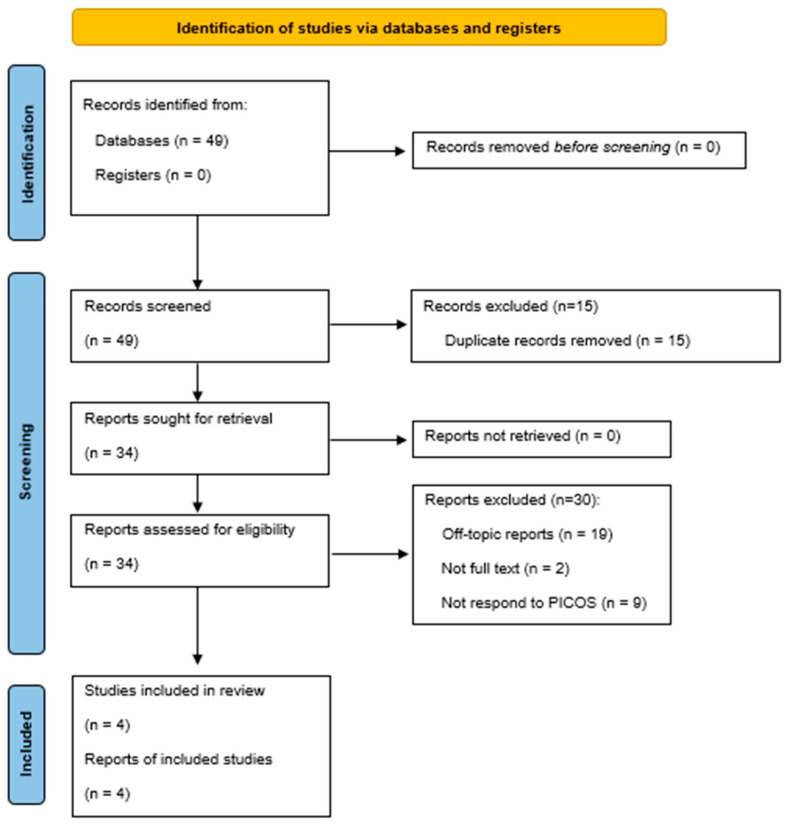
PRISMA flowchart of the included study.

The selection process is visualized in the PRISMA flow diagram (Figure 1).

### 2.4. Quality Assessment

Two reviewers (S.S. and V.R) evaluated the risk of bias through the Robins-I tool. A third reviewer (M.C.) was consulted to resolve any disagreements until consensus was obtained.

The Robins-I tool was used to assess the risk of bias in non-randomized intervention studies (NRSIs). It allows us to evaluate the risk of bias through a structured approach to evaluate potential biases, using seven domains: confounding, participant selection, intervention classification, deviations from intended interventions, missing data, outcome measurement, and selection of reported results.

Two trained reviewers independently assessed each study, following the ROBINS-I guidelines. This process ensured objectivity and consistency in the evaluations. The ROBINS-I assessment provided a comprehensive analysis of potential biases, highlighting both the strengths and limitations of the evidence base. This, in turn, informed the interpretation of results and supported more reliable conclusions based on the available data.

## 3. Results

### 3.1. Study Characteristics

The search provided a total of 49 articles including 15 articles from PubMed, 7 articles from Scopus, 21 articles from Google Scholar, and 6 articles from Web of Science. The Boolean operator “NOT” was used to exclude articles written not in English language, according to exclusion criteria.

Due to their duplication, 15 articles were removed. Then, 34 articles were assessed for eligibility. Of these, 30 were excluded because 19 were off-topic, the full text was unavailable for 2, and 9 did not meet the PICOS criteria. The large number of excluded studies mainly reflects the limited relevance of many articles to the specific field of OMFS, the absence of original data, and the strict application of PICOS criteria. Additionally, the recent introduction of LLM-based chatbots (since late 2022) means that only a few primary studies have been published to date. Consequently, four articles were included in this revision, as illustrated by the PRISMA 2020 flowchart (Figure 1 and Table 2). The four articles that met the inclusion criteria, all were published in 2024; in total, 2/4 (50%) articles assessed how effectively these chatbots performed in answering patients’ inquiries about OMFS and 2/4 (50%) articles evaluated ChatGPT’s reliability and accuracy in responding to general dentists’ questions about OMFS. No articles from 2025 were included because, as of 8 April 2025, no publications meeting our defined PICOS criteria had been identified.

While all four studies shared a focus on LLM-based chatbot applications within OMFS, marked heterogeneity was present in several areas:

- Unit of analysis: Two studies assessed chatbot responses to layperson queries (patient-level communication), while the other two analyzed responses to specialized, case-based, or procedural questions (clinician-level interaction).
- Methodological approaches: These ranged from structured question sets rated by expert panels (e.g., five-point or three-point Likert scales, Global Quality Scale) to repeated simulations using large batches of identical prompts (e.g., 30 × 30 input permutations).
- LLM platforms and versions: Tools evaluated included GPT-3.5, GPT-4 (OpenAI), Bing (Microsoft), Bard (Google), and Claude Instant (Anthropic), with performance varying significantly across models and use cases.
- Sample sizes and study contexts: Expert rates ranged from 2 to 10 in number, and the number of evaluated chatbot responses ranged from 20 to 900 across studies.

The inter-rater reliability between the two independent reviewers was substantial, with a Cohen’s kappa coefficient of κ = 0.76, indicating good agreement in the study selection process. Discrepancies that arose were resolved through consensus with the third reviewer, ensuring a rigorous and unbiased screening process.

### 3.2. Main Findings

This synthesis involves multi-level analysis. At the broader level, studies are compared based on methodology, context, and objectives. Within each study, chatbot-generated responses are analyzed to assess the performance of specific LLMs, enabling a comprehensive understanding of their effectiveness in clinical settings.

The included studies (summarized in Table 2) evaluated LLM-based chatbots such as ChatGPT versions 3.5 and 4, Bing, Bard, and Claude Instant.

#### 3.2.1. Chatbot Powered by LLMs and Patients

In the comparative study of Acar 2024 [21], the accuracy and completeness of popular LLM-based chatbots—ChatGPT 3.5, Microsoft Bing, and Google Bard—were evaluated by ten experts in oral and maxillofacial surgery. The evaluation focused on the chatbots’ responses to patient questions about common oral surgery complications, such as pain, numbness, and bleeding following tooth extraction and dental implants. ChatGPT demonstrated superior accuracy, with a mean score of 1.4000 ± 0.15986 on the five-point Likert scale, significantly outperforming Microsoft Bing (1.8750 ± 0.18143) and Google Bard (2.0500 ± 0.12472) (*p* < 0.001). Additionally, ChatGPT achieved a higher Global Quality Scale (GQS) score of 4.4200 ± 0.30111, which assesses the quality of information, compared to Bing (3.7550 ± 0.28621) and Bard (3.5250 ± 0.22392) (*p* < 0.001). Bing also showed a statistically significant advantage over Bard in GQS scores. These findings suggest that natural language processing (NLP) technologies, like chatbot platforms, have promising potential as consultative tools in oral surgery. They could assist in diagnostics, treatment planning, and postoperative care by efficiently processing information and responding to specific queries. However, the study also emphasized that the complexity of clinical scenarios limits AI’s ability to fully replace human judgment [21].

Cai et al. 2024 [24] explored the application of GPT-4 for postoperative patient follow-up in oral surgery. The model was assessed for its ability to manage patient communication, monitor healing, and answer common postoperative questions. Three experts in oral and maxillofacial surgery analyzed the accuracy of responses to thirty frequently asked questions, grouped into the following categories: (A) follow-up after simple or surgical tooth extraction, (B) follow-up after other oral surgeries, and (C) miscellaneous follow-up queries. The study also highlighted GPT-4’s superior capacity to detect patient anxiety and address emotional concerns, not only by providing accurate medical advice but also by offering empathetic reassurance—an improvement over ChatGPT-3.5. All response categories were deemed medically accurate and relevant, with GPT-4 outperforming its predecessor. The results indicate that GPT-4 could help reduce healthcare providers’ workload by enhancing patient communication and delivering timely answers to common questions. Nonetheless, patient feedback pointed out that the absence of direct personal interaction may negatively affect overall satisfaction [24].

#### 3.2.2. Chatbot Powered by LLMs and Specialties of Sectors or Generic Dentistry

The study by Suárez et al. 2024 [22] investigated ChatGPT’s potential as a virtual assistant in oral surgery, supporting clinical decisions and real-time consultation during procedures. ChatGPT-4 posed 30 oral surgery-related questions, each repeated 30 times, resulting in a total of 900 responses. Two surgeons evaluated these answers using a three-point Likert scale, following criteria from the Spanish Society of Oral Surgery. The accuracy of responses varied widely across questions, ranging from 0% to 100%, with an overall correctness rate of 71.7% (95% confidence interval: 68.9–74.6%). These findings suggest that ChatGPT could aid surgeons by delivering rapid, data-driven information and advice. However, the study also emphasized that the quality of prompts influences response accuracy, and human oversight remains crucial for safe and effective application [22].

Azadi et al.’s 2024 study [25] compared multiple LLM-based chatbots in clinical decision-making scenarios within OMFS. Three expert evaluators assessed answers from Bard, GPT-3.5, GPT-4, Claude-Instant, and Bing, which correctly responded to 34%, 36%, 38%, 38%, and 26% of questions, respectively. For open-ended questions, GPT-4 achieved the highest number of top ratings (“4” or “5”), while Bing received the lowest scores (“1” or “2”). The results indicate that although some AI models provide accurate answers, performance varies considerably depending on question phrasing and complexity. While AI can offer relevant insights, it does not yet match the depth of analysis provided by human clinical experts [25].

### 3.3. Comparative Analysis of Results

The comparative analysis of the four included studies highlights significant convergences and divergences regarding the use of chatbots based on LLMs in the field of OMFS. The differences mainly concern the application purpose (patient communication vs. clinician support), the versions of the model used (GPT-3.5 vs. GPT-4), the evaluation methodology, and the results obtained in terms of accuracy, quality, and reliability.

From the perspective of patient interaction, the works by Acar (2024) [21] and Cai et al. (2024) [24] demonstrated that ChatGPT, especially the newer GPT-4, delivers accurate, thorough, and reassuring responses to questions about postoperative complications and recovery. Acar’s study showed that ChatGPT-3.5 outperformed Bing and Bard in both accuracy (Likert 1.4 ± 0.15 vs. 1.87 and 2.05) and clarity (GQS 4.42 ± 0.30). Meanwhile, Cai highlighted GPT-4’s ability to perceive patient emotions and respond empathetically and appropriately, supporting its potential role in postoperative follow-up.

In the professional and decision-making domain, Suárez et al. (2024) [22] and Azadi et al. (2024) [25] evaluated LLM-based chatbots as tools for supporting professional judgment. Suárez reported an overall accuracy of 71.7% for ChatGPT-4 across 900 clinical questions, noting that response quality depends heavily on prompt wording and question complexity. Azadi’s comparison showed GPT-4 and Claude Instant achieving the highest correct-answer rates (38%), while Bing performed less well (26%). The study also pointed out significant variability in the performance of LLM-based chatbots, indicating that these models are not yet reliable for independent use in complex clinical situations.

Overall, the most accurate model across studies was GPT-4, consistently outperforming previous versions and other LLM-based chatbots in both patient communication and clinical decision support contexts. Typical accuracy ranged from approximately 70% to 75% in clinical question answering, while clarity and quality scores were higher for GPT models compared to competitors. These trends highlight ongoing improvements in LLM capabilities but also emphasize variability depending on use case and question complexity.

A common conclusion from all studies is that AI currently serves as a valuable adjunct to clinical practice and doctor–patient communication but cannot replace professional expertise. The performance improvements seen between GPT-3.5 and GPT-4 highlight rapid algorithmic advancements, underscoring the need for continuous evaluation tailored to specific use cases.

### 3.4. Quality Assessment and Risk of Bias

**Figure 2:**
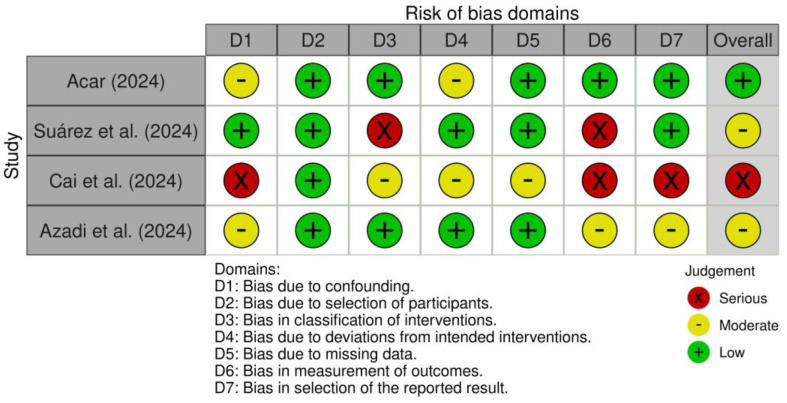
ROBINS-I tool for risk of bias for studies analyzed [21,22,24,25].

The risk of bias was evaluated using the ROBINS-I tool, shown in Figure 2. Regarding confounding bias (D1), one study (Suárez et al. 2024 [22]) showed a low risk of bias. The bias due to selection of participants (D2) demonstrated a low risk of bias in all studies analyzed. Bias arising from classification of interventions (D3) was rated as low risk in Acar’s 2024 [21] and Azadi et al.’s 2024 studies [25], serious in the Suárez et al.’s 2024 [22] study, and moderate in Cai et al.’s 2024 study [24]. Bias in the deviations from intended investigations (D4) was consistently low in two studies and moderate in the other two studies. For missing data (D5), three studies analyzed showed a low risk of bias, and one study presented a moderate risk of bias. The distortion in measuring results (D6) is due to the exclusive use of LS, which are based on subjective assessments that may not accurately reflect reality, to evaluate the accuracy of the analyzed LLM-based chatbots (serious risk of bias for the Suárez et al. 2024 [22] and Cai et al. 2024 [24] studies). In the other two studies, more accredited rating scales are used, thus reducing the risk of bias to moderate for the Azadi et al. 2024 [25] study and low for the Acar 2024 [21] study. A low risk of bias in the selection of the reported result (D7) was indicated in two studies (Acar 2024 [21] and Suarez et al. 2024 [22]), a moderate risk in the Azadi et al. 2024 [25] study, and a serious risk in the Cai et al. 2024 [24] study.

Overall, the studies were judged as follows: serious risk of bias for Cai et al. 2024, moderate risk for Acar and Suárez et al.’s studies [21,22], and low risk for Azadi et al. 2024 [25].

## 4. Discussion

The use of AI in the medical sector, including dentistry, has become a highly debated topic. Emerging evidence highlights the potential of AI systems—especially those powered by LLMs—to enhance clinical efficiency, reduce the practitioner workload, and support decision-making. In standardized, routine scenarios, LLM-based chatbots excel by providing rapid answers to common clinical queries, assisting in treatment planning, automating repetitive tasks, and standardizing patient communication before and after surgery [26]. However, LLM-based chatbots’ ability to replace human clinical judgment remains limited, particularly in complex or non-standardized cases where accuracy and contextual understanding decrease as complexity rises [21,27]. Additionally, the quality of LLM-based chatbots’ output is highly dependent on prompt formulation, which introduces variability and potential for error. From the patient perspective, while LLM-based chatbots offer constant accessibility, concerns persist regarding the lack of human interaction and emotional reassurance—an especially relevant factor in OMFS, which often involves anxiety-inducing procedures [28,29]. Although AI can recognize emotional cues like tone of voice or facial expressions, it lacks the contextual understanding needed for genuine emotional support—especially in sensitive areas like OMFS. Nonetheless, LLM-based chatbots have proven effective in promoting health behavior change [1] and supporting treatment adherence by providing instant, non-judgmental communication spaces, as shown in recent systematic reviews in healthcare and orthodontics [30].

This review should be considered an initial synthesis of the evidence, reflecting the nascent state of research on LLM-based chatbots in OMFS. The limited number of available studies and their heterogeneity restrict the generalizability of conclusions. Nonetheless, this exploratory analysis offers valuable insights and underscores the urgent need for larger, methodologically rigorous studies to better understand the potential and limitations of this technology. Comparing different studies allowed us to identify key trends, such as the higher accuracy of certain models in standard cases, but also variability in results and a lack of homogeneous data that make direct comparison difficult. The summary table (Table 3) facilitates an understanding of clinical, operational, and patient management aspects where AI can intervene, while also emphasizing current limitations.

Our findings reinforce the role of AI as a complementary tool rather than a replacement for clinicians. Studies by Acar’s in 2024 [21] and Suárez et al. in 2024 [22] highlight LLM-based chatbots’ value in assisting clinical tasks and delivering quick information, while acknowledging its limits in adapting to unforeseen surgical developments. In 2024, Azadi et al. [25] reported mixed accuracy, emphasizing the need for cautious interpretation.

LLM-based chatbots can automate administrative tasks and support clinicians in patient management, including pre- and post-operative care and medical documentation. Studies (Cai et al. 2024 [24], Acar 2024 [21]) show that LLM-based chatbots may reduce workload in post-operative care. However, patients often express concern over the lack of direct human contact (Cai et al. 2024 [24] and Suárez et al. 2024 [22]). OMFS involves emotionally sensitive situations where AI, despite its accuracy, cannot provide the emotional understanding and reassurance that human surgeons offer. Thus, integrating AI in OMFS requires balancing efficiency with essential human interaction [31].

Beyond OMFS, LLM chatbots have shown benefits in orthodontics and periodontology, improving patient education and treatment adherence. A recent randomized controlled trial demonstrated that LLM-based chatbots can significantly enhance patient education, improve understanding of orthodontic treatment procedures, and positively influence patient compliance in terms of adherence to oral hygiene maneuvers [30]. In periodontology, chatbots powered by LLM have been employed to support patient communication, providing explanations of periodontal conditions and reinforcing home care recommendations [32]. However, limitations in surgical contexts highlight the need for specialty-specific strategies, considering that OMFS involves acute, unpredictable procedures, requiring immediate decisions, clinical sensitivity, and emotional intelligence—less critical factors in orthodontics or prosthodontics [21,22].

The screening and selection of studies were carried out independently by two reviewers, with any disagreements resolved by consultation with a third reviewer. Inter-rater reliability was assessed using Cohen’s kappa coefficient, resulting in a value of 0.78, which indicates substantial agreement and strengthens the credibility of the study selection process. This approach helped minimize selection bias and supports the robustness of the review’s conclusions.

However, several limitations should be acknowledged:

- The number of eligible primary studies was relatively small, mainly due to the recent introduction of large language model (LLM)-based conversational agents in medical and dental contexts. This limited sample may reduce the precision of the results and the generalizability of the conclusions.
- Significant heterogeneity was observed among the included studies regarding study design, evaluation metrics, and clinical settings, which limited the comparability of outcomes and prevented meta-analytical synthesis.
- The review was restricted to studies published in English and did not include gray literature, potentially introducing language and publication biases.
- The review did not incorporate expert opinions or clinical perspectives, which could have provided valuable contextual insight into the findings.
- Considering the rapid pace of technological advancement and the continuous emergence of new evidence, it is essential that this review be regularly updated to remain current, relevant, and aligned with the evolving landscape of AI applications in OMFS.

Future research should not only refine the accuracy and training of models on specific datasets but also include prospective clinical trials in real-world settings to evaluate the actual effectiveness and impact of LLM-based chatbots in daily clinical practice in OMFS. At the same time, it is crucial to develop robust frameworks for safe and ethical implementation, encompassing clinical supervision, privacy protection, medico-legal responsibility, and informed consent. An interdisciplinary collaboration among clinicians, AI developers, and regulators will be essential to guide these advancements responsibly and sustainably.

## 5. Conclusions

The synthesis of current evidence highlights the need for a balance between AI and clinical expertise. LLM-based chatbots show promising potential as support tools for healthcare professionals, helping to improve efficiency, accuracy, and decision-making in routine OMFS cases. However, their use still presents significant limitations in patient interaction, particularly in their ability to provide genuine emotional support—an essential component of surgical care. Across studies, the most consistent finding is that while LLM chatbots perform reliably in standardized, straightforward clinical scenarios, their accuracy and contextual understanding decline notably in complex or non-standardized cases. Despite the limited scope and heterogeneity of existing studies, there is a clear need for future research that is larger in scale, methodologically rigorous, and standardized. Such studies are essential to produce more robust and generalizable conclusions. Furthermore, future investigations should focus on the practical integration of LLM-based chatbots into clinical workflows, supported by qualitative insights from oral surgeons to capture the nuanced realities of surgical practice.

## Figures and Tables

**Table 1:** Search strategy.

**PubMed**
((“Oral Surgical Procedures” [MeSH Terms] OR “oral surgery” [All Fields] OR “dental surgery” [All Fields] OR “maxillofacial surgery” [All Fields]) AND (“large language models” [All Fields] OR “LLM” [All Fields] OR “ChatGPT” [All Fields] OR “GPT” [All Fields] OR “generative pre-trained transformer” [All Fields] OR “pretraining language model” [All Fields] OR “AI language model” [All Fields] OR “chatbot” [All Fields] OR “natural language processing” [MeSH Terms] OR “natural language processing” [All Fields] OR “NLP” [All Fields]))
**Scopus**
TITLE-ABS-KEY ((“oral surgery” OR “dental surgery” OR “maxillofacial surgery”) AND (“large language model” OR LLM OR ChatGPT OR GPT OR “generative pre-trained transformer” OR “pretraining language model” OR “AI language model” OR chatbot OR “natural language processing” OR NLP))
**Web of Science**
TS = (“oral surgery” OR “dental surgery” OR “maxillofacial surgery”) AND TS = (“large language model” OR LLM OR ChatGPT OR GPT OR “generative pre-trained transformer” OR “pretraining language model” OR “AI language model” OR chatbot OR “natural language processing” OR NLP)
**Google Scholar**
“oral surgery” OR “dental surgery” OR “maxillofacial surgery” AND “large language model” OR LLM OR ChatGPT OR GPT OR “generative pre-trained transformer” OR “AI language model” OR chatbot OR “natural language processing” OR NLP

**Table 2:** Summary of studies that were included in the systematic review.

Author	Year	Country	Title	Application Area	LLM Tool(s))	Sample Sizes	Methods	Scale Assessing	Key Results	Conclusion
Acar [21]	2024	Turkey	Can natural language processing serve as a consultant in oral surgery	Clinical Q&A in oral surgery	ChatGPT 3.5 (OpenAI), Bing(Microsoft), Bard (Google)	10 experts, 20 questions	Experts submitted 20 clinical questions to 3 chatbots	Five-point Likert Scale (accuracy/completeness of responses); Global Quality Scale (clarity of responses)	ChatGPT outperformed Bing and Bard in both accuracy and clarity, with statistically significant differences (*p* < 0.001).	ChatGPT provided more accurate and clear responses
Suárez et al. [22]	2024	Spain	Beyond the Scalpel: Assessing ChatGPT’s potential as an auxiliary intelligent virtual assistant in oral surgery	Clinical decision-making support in oral surgery	ChatGPT-4 (OpenAI)	2 experts, 30 × 30 = 900 replies	Two oral surgeons evaluated ChatGPT-4’s accuracy and reliability as a virtual assistant for clinical decision-making in oral surgery.	Three-point Likert Scake (accuracy)	Accuracy reached 71.7%, with expert judgment consistency ranging from moderate to nearly perfect.	ChatGPT-4, could have potential as virtual assistance in oral surgery.
Cai et al. [24]	2024	China	Exploring the use of ChatGPT/GPT-4 for patient follow-up after oral surgeries	Patient follow-up and reassurance after oral surgery	ChatGPT-3.5 OpenAI)/GPT-4 (OpenAI)	3 experts, 30 questions	Three oral and maxillofacial surgeons tested chatbot replies to common follow-up questions	Score 0–10 (accuracy and advice quality)	ChatGPT-3.5/GPT-4 scored perfectly for medical accuracy and recommendation rationality, also effectively sensing and reassuring patient emotions.	ChatGPT/GPT-4 could be used for patient follow-up after oral surgeries.
Azadi et al. [25]	2024	Iran	Evaluation of AI-generated responses by different artificial intelligence chatbots to the clinical decision-making case-based questions in oral and maxillofacial surgery	Clinical decision-making with case-based questions in OMFS	GPT-3.5 (OpenAI), GPT-4 (OpenAi), Claude-Instant (Anthropic), Bing (Microsoft), Bard (Google)	3 experts, 50 case-based questions in multiple-choice (MCQ) and open-ended (OQ) formats.	A group of 3 board-certified oral and maxillofacial surgeons evaluated answers to MCQ and open-ended Qs	Modified Global Quality Scale (GQS).	Bard, GPT-3.5, GPT-4, Claude-Instant, and Bing answered 34%, 36%, 38%, 38%, and 26% of questions correctly. GPT-4 had the highest scores (“4” or “5”) on open-ended questions, while Bing had the most low scores (“1” or “2”)	LLM-based chatbots are not yet reliable consultants for clinical decision-making.

**Table 3:** Summary of the strengths and limitations of LLM-based chatbots reported in studies related to OMFS.

Aspect	Documented Advantages	Highlighted Limitations	Main Sources
Clinical Decision Support	Quick responses to common clinical questions; assistance in treatment planning	Accuracy not guaranteed in complex or non-standardized cases	Acar 2024 [21], Azadi 2024 [25]
Operational Efficiency	Reduction in workload; automation of repetitive tasks	Dependence on prompt quality and risk of poorly contextualized information	Suárez 2024 [22], Cai 2024 [24]
Intraoperative Assistance	Potential real-time support in managing intraoperative complications	Lack of expert clinical judgment and inability to react to unforeseen variables	Suárez 2024 [22]
Patient Management (Pre/Post-op)	Standardized communication regarding surgical instructions and follow-up	Lack of empathy and inability to provide human reassurance	Cai 2024 [24], Suárez 2024 [22]
Response Accuracy	High for frequently asked questions and standard cases	Low consistency in complex scenarios; potential contextual errors	Azadi 2024 [25]
Patient Acceptability	Constant accessibility and availability	Concerns about the lack of human contact and personalization	—

## Data Availability

No new data were created or analyzed in this study.

## Abbreviations

The following abbreviations are used in this manuscript:

AI	Artificial Intelligence
LLMs	Large Language Models
NLP	Natural Language Processing
PECO	Population, Exposure, Comparator, Outcomes
PRISMA	Preferred Reporting Items for Systematic Reviews and Meta-Analyses
ROBINS-I	Risk Of Bias in Non-Randomized Studies of Interventions
USMLE	United States Medical Licensing Examination
NRSIs	Non-Randomized Intervention Studies
LS	Likert Scale
GQS	Global Quality Scale
OMFS	Oral and Maxillofacial Surgery
